# The Application of Real-Time PCR Technique to Detect Rare Cell Clones with Primary T790M Substitution of *EGFR* Gene in Metastases of Non-small Cell Lung Cancer to Central Nervous System in Chemotherapy Naive Patients

**DOI:** 10.1007/s12253-014-9778-6

**Published:** 2014-05-03

**Authors:** Tomasz Powrózek, Paweł Krawczyk, Bożena Jarosz, Radosław Mlak, Kamila Wojas-Krawczyk, Marek Sawicki, Dariusz Stencel, Tomasz Trojanowski, Janusz Milanowski

**Affiliations:** 1 Pneumonology, Oncology and Allergology Department, Medical University of Lublin, Lublin, Poland; 2 Neurosurgery and Pediatric Neurosurgery Department, Medical University of Lublin, Lublin, Poland; 3 Thoracic Surgery Department, Medical University of Lublin, Lublin, Poland; 4 Boehringer Ingelheim Poland, Warsaw, Poland; 5 Institute of Agricultural Medicine, Lublin, Poland; 6 Immunology and Genetics Laboratory, Pneumonology, Oncology and Allergology Department, Medical University of Lublin, Jaczewskiego 8, 20-954 Lublin, Poland

**Keywords:** Non-small cell lung cancer, *EGFR* gene, T790M mutation, Metastases to central nervous system, EGFR tyrosine kinase inhibitor

## Abstract

The time-limited efficacy of reversible EGFR-TKIs in patients with advanced non-small cell lung cancer (NSCLC) with *EGFR* gene activating mutations is associated with development of treatment resistance after some period of therapy. This resistance predominantly results from secondary mutations located in *EGFR* gene, especially T790M substitution. There is limited information available concerning the prevalence of primary T790M mutations in patients with metastatic NSCLC tumors before treatment with EGFR-TKIs. The aim of work was to assess the prevalence of de novo T790M mutations in *EGFR* gene in tissue samples from NSCLC metastatases in central nervous system (CNS) in both chemotherapy and EGFR-TKI naive NSCLC patients. We analyzed DNA samples isolated from paraffin-embedded tissue from CNS metastases for T790M mutations using real-time PCR and TaqMan probe against the T790M mutant sequence. The tissue samples were taken during palliative neurosurgery in 143 NSCLC patients. Amplification of the T790M-specific sequence was detected in 25 patients (17.5 %). The quantity of mutated DNA was less than 1 % in all samples with amplification, and in vast majority (20 patients, 14 % of all samples) it was even less that 0.1 %. In 5 patients (3.5 %) quantity of mutated DNA ranged from 0.1 to 1 % and true positive results of T790M mutation presence in these patients were most possible. Amplification of this sequence was not concurrent with common *EGFR* mutations and was not associated with sex, smoking status and pathological type of cancer. There is a possibility to detect the primary T790M mutation in brain metastases of NSCLC in EGFR-TKIs naïve patients.

## Introduction

Various genetic disorders, such as mutations as well as genome rearrangements in non-small-cell lung cancer (NSCLC) cells are currently the subject of intensive research. Whilst the detection of the genetic abnormalities could enable to develop the new target for different targeted therapies, the detailed descriptions of those changes could help to understand of mechanisms of resistance for already existing therapies. Genetic disorders in rare cell clones from heterogenic NSCLC tumors and their possible role in designing of molecular targeted therapy in the patients not responding for modern therapies are becoming of great interest.

T790M mutation in *EGFR* gene seems to be one of the most important genetic abnormalities involving the resistance for reversible epidermal growth factor receptor tyrosine kinases inhibitors (EGFR-TKI)—erlotinib and gefitinib in NSCLC patients [1–3]. T790M mutation is detected with highest prevalence in patients with common activating mutations of *EGFR* gene (L858R in exon 21 and deletions in exon 19) after long-term and initially effective treatment with EGFR-TKIs. Based on this finding it was considered that T790M is a secondary resistance mutation resulting from EGFR-TKIs therapy. Recently it was suggested that substitution in codon 790 of exon 20 *EGFR* gene could be visible primary in coincidence with other activating mutations of *EGFR* gene (5–30 % NSCLC patients) [4–6] and secondary, after primary *EGFR* disorders, mainly L858R mutation (app. 50 % patients) [2, 5–7].

Although T790M mutation plays a leading role in emergency of reversible EGFR-TKIs resistance, the mechanism of its appearance is still unclear. Whilst it was suggested, that T790M mutation is driven by EGFR-TKIs therapy, recently this is postulated, that this mutation is present at the very beginning in a rare clone of NSCLC cells even in EGFR-TKIs naive patient. The cells with T790M mutation are not detectable during primary *EGFR* status assessment due to very low sensitivity of available molecular biology methods. Then, as results of effective EGFR-TKI treatment, the larger clone with L858R mutation or deletions in exon 19 in *EGFR* gene is eliminated and treatment-resistant cell clones, containing wild type *EGFR* gene or T790M mutation can proliferate [3, 5, 6]. In those cases T790M mutations could be detectable in the same tumors containing L858R substitutions or deletions in exon 19, but on distinct cell clones.

Although there is an increasing knowledge about prevalence and detectability of activating mutations of *EGFR* gene in NSCLC metastatic changes in central nervous system (CNS), the primary mutations, connected with molecular targeted therapy resistance in patient with distant metastases remain unresolved [8, 9]. The aim of work was to assess the sensitive method, enabling detections of cells with T709M mutations, to estimate their percentage as well as to assess the prevalence of primary T790M mutation in metastatic NSCLC changes in CNS.

## Material and Method

To assess the prevalence of T790M mutation in exon 20 of *EGFR* gene we retrospectively analyzed the paraffin-embedded material from cancer tissue taken from CNS metastases from totally 143 NSCLC patients (99 male and 44 female) at the age ranged 38–81 (mean: 59.8 ± 8.8 years). The patients underwent neurosurgery palliative procedure in routine manner. The patients were chemotherapy as well as EGFR-TKI naïve.

Based on histological evaluation in 61 (42.6 %) patients adenocarcinoma was diagnosed, in 23 (16.1 %) patients squamous cell and in 21 (14.7 %) patients large cell carcinoma. In 38 (26.6 %) patients NSCLC remained “not otherwise specified” (Table 2). Median of overall survival (OS) since diagnose to death was 9.2 months (range: 0.1–78.2 months).

To isolate DNA samples from paraffin-embedded NSCLC tissue Qiamp DNA FFPE Tissue Kit (Qiagen, USA) was used. DNA concentration was measured in Biofotometr Plus spectrophotometer (Eppendorf, Germany). The substitution T790M in exon 20 in *EGFR* gene was assessed by real-time PCR using specific for mutated *EGFR* gene molecular probes TaqMan (Applied Biosystem). Real-time PCR was conducted in Eco Real-Time PCR System (Illumina) according to Mutation Detection Assays Protocol (Applied Biosystems), delivered by manufacturer of TaqMan® reagents. Additionally, for each sample the reference gene amplification was performed using molecular probes complementary for non-mutated *EGFR* gene part (positive intrinsic control). For negative control DNA isolated from peripheral blood leukocytes of healthy subjects was used.

Furthermore, in analyzed material the prevalence of L858R substitution in exon 21 and deletion in exon 19 of *EGFR* gene were assessed using the following techniques: PNA-LNA PCR Clamp (for both common *EGFR* gene mutations), ASP-PCR (for L858R substitution) and PCR and analysis of the length of amplified DNA chains (for deletions in exon 19). The presence of L858R mutation and deletion in exon 19 of *EGFR* gene were confirmed using direct sequencing method. This method was not used for the confirmation of T790M substitution due to its low sensitivity (requiring above >50 % cells with mutated DNA).

**Fig. 1:**
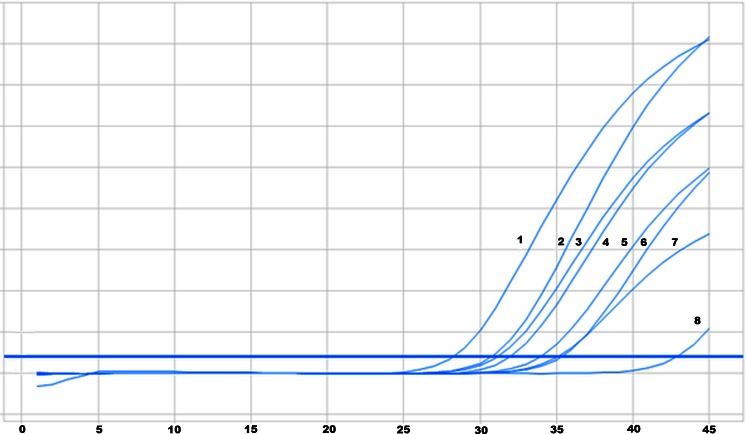
Amplification curves for DNA with T790M mutation diluted with control DNA and control DNA alone: 1–50 %, 2–25 %, 3–20 %, 4–10 %, 5–5 %, 6–2 %, 7–1 %, 8—control DNA

To assess the sensitivity of real-time PCR method used for detection of T790M substitution in *EGFR* gene the series of dilutions of DNA isolated from H1975 cell line containing substitutions T790M and L858R was prepared. Mutated DNA was diluted with DNA isolated from peripheral blood leukocytes from healthy subjects, which concentration was identical than mutated DNA. There were the following mutated DNA concentrations: 50, 25, 20, 10, 5, 2 and 1 %. The result of real-time PCR and amplification curves for analyzed dilutions are presented on Fig. 1.

**Fig. 2:**
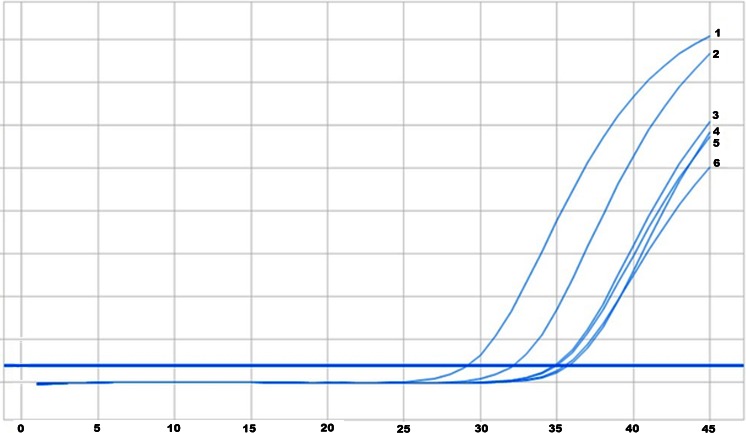
Amplification curves for DNA with T790M mutation diluted with control DNA and control DNA alone: 1–10 %, 2–5 %, 3–2 %, 4–1 %, 5–0,1 %, 6–<0,1 %

Again, in the second part of the experiment the series of dilutions of DNA isolated from H1975 cell line was prepared, but with higher DNA dilutions than in the first part: 10 %, 5 %, 2 %, 1 %, 0,1 %, <0,1 %. The result of real-time PCR and amplification curves for analyzed dilutions are presented on Fig. 2.

## Results

Nine most common activating *EGFR* gene mutations (6.29 %) were detected in CNS metastases of NSCLC: 3 deletions in exon 19 (2.1 %) and 6 substitutions L858R (4.2 %). In none of them the amplification of sequence containing the T790M mutation was detected (no signal from T790M mutation- specific probe).

**Fig. 3:**
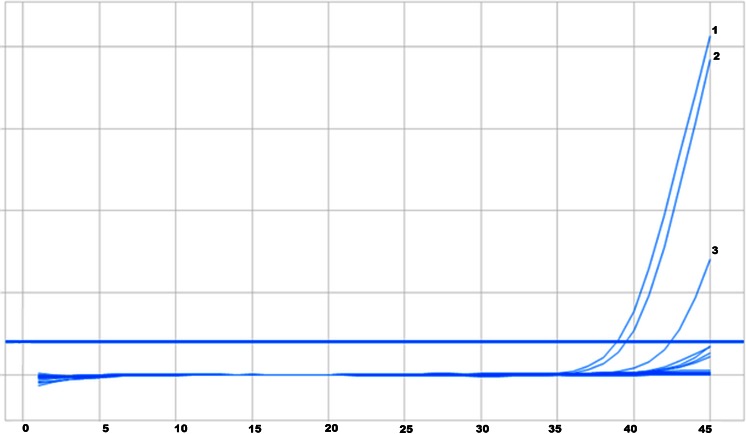
Amplification curves for DNA with T790M mutation: curves 1 and 2 – patients, curve 3—control DNA

Based on real-time PCR results the presence of amplification DNA product containing T790M mutation was revealed in 34 patients (23.8 % of evaluated group). In each case the amplification of reference gene was also observed (positive intrinsic control). During 45 cycles-real-time PCR the amplification of negative control (non-mutated DNA) was shown, usually after 43, cycle of PCR reaction, which possible resulted from unspecific binding of the probe to analyzed DNA sample. Taken the number of cycles, after which amplification of negative control occurred as a cut-off point between false positive and false negative results, the appearance of amplification product was judged as unspecific in 9 patients (6.3 %). Figure 3 presents the result of T790M mutation in 2 patients with presence of amplification product of DNA sequence containing T790M mutation in *EGFR* gene.

During second part of the experiment all samples were tested for T790M substitution with lowering number of reaction cycles to 42. Because the negative control was usually amplified after 43. cycle we determine cut off point in 42.cycle. Upon those conditions the amplification product of mutated DNA sequence was detected in 25 patients (17.5 %), whereas the amplification of negative control was not observed.

During the next part the percentage of copies of mutated *EGFR* gene with T790M mutation among wild-type DNA copies was estimated. Firstly, the amplification curves for the series of dilutions of DNA from H1975 cell line (used for calibration) were compared with amplification curves for cases with detected specific product of reaction for T790M mutation. Secondly, estimated content of mutated DNA in analyzed samples was calculated, using the formula:

$$ \%\ \mathrm{mutated}\ \mathrm{DNA}=1/{2}^{\varDelta \varDelta Ct}\ast 100\% $$

where: ∆∆Ct = ∆Ct (analyzed sample) − ∆Ct (calibrator). To calculate ∆Ct for analyzed sample and calibrator the difference between threshold values of real-time PCR (Ct) was calculated, using the formulas:

$$ \begin{array}{c}\hfill \varDelta \mathrm{Ct}\left(\mathrm{analyzed}\ \mathrm{sample}\right)=\mathrm{Ct}\ \mathrm{of}\ \mathrm{analyzed}\ \mathrm{gene}-\mathrm{Ct}\ \mathrm{of}\ \mathrm{reference}\ \mathrm{gene}\hfill \\ {}\hfill \varDelta \mathrm{Ct}\left(\mathrm{calibrator}\right)=\mathrm{Ct}\ \mathrm{of}\ \mathrm{calibrator}-\mathrm{Ct}\ \mathrm{of}\ \mathrm{reference}\ \mathrm{gene}\hfill \end{array} $$

Based on mathematical analysis, the content of mutated DNA in all positive samples was assessed as <1 % (Table 1). Table 2 presents estimated content of mutated DNA in analyzed material.

**Table 1:** Estimated content of DNA with T790M mutation in analyzed material

Content of mutated DNA in analyzed material	Number of patients with amplification of DNA sequence containing T790M mutation
<1 %	25
0.5–1 %	1
0.2–0.5 %	1
0.1–0.2 %	3
<0.1 %	20

Additionally it was found, that prevalence of real-time PCR products specific for DNA sequence with T790M mutation was similar in male and female patients, smokers and non-smokers and across different histological NSCLC types (Table 2).

**Table 2:** The prevalence of amplification of mutated *EGFR* gene fragment containing T790M mutation in CNS metastases of NSCLC

Clinical measures	Prevalence of T790M mutation (<1 % of mutated DNA)	Prevalence of T790M mutation (1–0.1 % of mutated DNA)	Prevalence of T790M mutation (less than 0.1 % of mutated DNA)
Total cases (*n* = 143)	25 (17.5 %)	5 (3.5 %)	20 (14 %)
NSCLC-NOS (*n* = 38)	10 (26.4 %)	2 (5.3 %)	8 (21.1 %)
Adenocarcinoma (*n* = 61)	8 (13.1 %)	1 (1.6 %)	7 (11.5 %)
Squamous cancer (*n* = 23)	4 (17.4 %)	0	4 (17.4 %)
Large-cell cancer (*n* = 21)	3 (14.2 %)	2 (9.5 %)	1 (4.7 %)
Sex			
Female (*n* = 44)	9 (20.5 %)	1 (2.3 %)	8 (18.2 %)
Male (*n* = 99)	16 (16.1 %)	4 (4 %)	12 (12.1 %)
Smoking status			
Smokers (*n* = 86)	17 (19.8 %)	3 (3.5 %)	14 (16.3 %)
Non-smokers (*n* = 32)	4 (12.5 %)	1 (3.1 %)	3 (9.4 %)
Lack of data (*n* = 25)	4 (16 %)	0	4 (16 %)

## Discussion

The role of T790M mutation, particularly its presence in patients before EGFR-TKIs treatment is still controversial being a subject of intensive discussions. Mechanism of achieving resistance to erlotinib and gefitinib remains unclear. The recent reports confirmed, that T790M substitution could be detected in EGFR-TKIs naïve patients independently of other *EGFR* gene mutations (predominantly L858R substitution and deletions in exon 19). This finding supports the observations, that coincidence of driving mutations is exquisitely infrequent.

Clone of cancer cells with some of driving mutations could be very small in heterogenic tumors, thus it could not be detected by methods like direct DNA sequencing. During EGFR-TKIs therapy the majority of cells with activating *EGFR* mutations undergo massive apoptosis leading to exposing and predominance of treatment-resistant subpopulation, e.g. with T790M mutation, which could be easier detected with standard molecular biology methods [3, 5].

The development of molecular biology methods, especially increased sensitivity of detection, supports mentioned above assumptions. Maheswaran et al. assessed tissue material from 26 EGFR-TKIs naïve patients with common activating *EGFR* gene mutation for T790M mutation. Using of SARMS-PCR technique with Scorpion probes in material with low content of mutated DNA allowed detecting substitutions in 10 patients (38 % analyzed sample). Appearance of product amplification after high number of cycles of PCR reaction used in this trial suggests, that mutation is present only in small tumor cells clone. Additionally, the sequencing of one of the PCR reaction products identified only one DNA copy with T790M mutation among 500 alleles of wild-type *EGFR* gene [4].

Based on this findings showing the presence of T790M mutation in very limited cancer cell clones, for detection of exon 20 of EGFR gene mutations Oh et al. used very sensitive PNA Clamp PCR (peptide-nucleic acid-clamp PCR) method, allowing detection of mutation even if it is in less than 1 % mutated cells. The mutation was revealed in 12 of 147 untreated patients (8.2 % of analyzed sample). However it was unselected group according to histological findings and *EGFR* gene status [6].

The very recent studies of Su et al. confirmed the theory how the T790M mutation becomes detectable. To confirm this mutation the authors used very sensitive MALDI-TOF MS (highly sensitivity matrix-assisted laser desorption ionization-time of flight mass spectrometry) method and next-generation sequencing. Using very sensitive methods made possible estimation of prevalence of T790M mutation in patients before and after cessation of EGFR-TKIs therapy. Mutation was detected in 27 among 107 EGFR-TKIs naive patients (25.2 %), whereas direct sequencing method led to detection of only 3 patients with T790M substitution (2.8 %). In group of 73 EGFR-TKIs treated patients T790M mutations were detected in 23 patients (31.5 %), whereas using direct sequencing method only in 2 patients (2.7 %). Additionally 12 samples from patients after discontinuation of EGFR-TKIs therapy due to progression were assessed. T790M mutation was confirmed in up to 10 patients (83.3 %). The study results support the conclusion, that primary T790M mutation is even more common than previously assumed. Additionally the authors concluded, that percentage of cells with primary T790M substitution is lower than in tumors after EGFR-TKIs therapy [5].

Noteworthy, according to different researchers, percentage of patients with de novo detected T790M mutation ranges widely from 1 to 38 %, what is possible depending on sensitivity of methods used for detection and sample selection [4–6, 10]. Furthermore, up to now it was conducted any study worldwide analyzing primary T790M mutations in NSCLC metastatic tumors to CNS or to other organs.

Our findings confirm, that primary T790M mutation is more common in NSCLC patients than it was suggested previously and that it could be detected in NSCLC metastatic changes in CNS. Detection of amplification product for mutated DNA sequence with T790M mutation was possible using real-time PCR—the most frequently used molecular biology method for diagnostic purposes. Furthermore, the study involved unselected population regarding histological finding and smoking status. It should be highlighted, that in each sample with amplification of *EGFR* gene region with T790M mutation the content of mutated DNA was less than 1 %, and in majority of cases (80 %) it was even below 0.1 %. Firstly, it seems to be impossible to repeat our finding using neither direct DNA sequencing method nor routine methods for in vitro diagnostics (it is assumed, that sensitivity of those methods should not allow to detect mutations when DNA content is below 10 %). Secondly, our finding should be treated with caution because they are very difficult to verify. It appears that the results of detection of T790M mutation are considerable within the range of DNA content between 0.1 and 1 % of analyzed material. Taking into consideration the fluorescence appearance from T790M mutation-specific probe even using wild-type DNA for real-time PCR, reliability of results from samples with DNA content below 0.1 % could be questioned (PCR products misamplified by polymerase). According to this it should be noted, that in our study, similarly to Maheswaran et al. study, we used high number of PCR cycles, after which amplification product was detectable. It is not routinely used for *EGFR* gene mutation diagnostic in clinical practice to avoid false positive results. Therefore, we are not sure that results below 0.1 % of mutated DNA were valid.

The possibility of detection of T790M mutation in EGFR-TKIs naive patients forces to expand the diagnostics of *EGFR* gene status during qualification for erlotinib or gefitinib treatment and to consider the new therapy modalities in carriers of this mutation. The new therapy methods, which could be effective in patients with T790M mutation includes irreversible EGFR-TKIs (afatinib, neratinib). In LUX-Lung 1 study in patients after failure of erlotinib or gefitinib treatment with afatinib led to partial responses and prolongation of progression-free survival compared to placebo to 3,3 months [11, 12]. Recently the studies are conducted with combining of irreversible EGFR-TKI and monoclonal antibody against extracellular domain of EGFR – cetuximab. In study conducted by Janjigian et al. all patients treated with afatinib and cetuximab achieved disease control, and part of them (36 %) partial response. Additionally, 4 of 13 patients (31 %) with T790M mutations gained confirmed partial response after combined therapy with monoclonal antibody and irreversible EGFR-TKI [13].

Currently, reversible EGFR-TKIs therapy is not contraindicated in carriers of T790M mutations. Furthermore, this mutation as well as other driving *EGFR* gene mutations, could be favorable predictive factor for EGFR-TKIs therapy. Recent studies indicated, that substitution T790M in EGFR-TKIs naïve patients could be connected to better prognosis and longer time to EGFR-TKIs therapy failure. In patients with T790M mutation median time do therapy failure was longer (9 months) than in patients without mutations (7 months). Additionally, median progression free survival was even longer in patients with higher percentage of mutated T790M allele than in patients with lower number of *EGFR* gene copies with T790M mutation [14]. Some reports deny the role of T790M mutation in carcinogenesis, since this mutation is more common in advanced NSCLC than in early stage tumors [10].
